# Expanding our Understanding of Atypical Antipsychotics: Acute Urinary Retention Secondary to Olanzapine

**DOI:** 10.1155/2020/6157548

**Published:** 2020-09-09

**Authors:** Hannah Mirzakhani, Mariam Rahim, Jacob Mathew

**Affiliations:** Department of Medicine, Parkview Medical Center, Pueblo, CO 81003, USA

## Abstract

Antipsychotics are considered the most efficacious drugs in the treatment of schizophrenia and other psychotic disorders. While clinicians monitor for the onset of extrapyramidal symptoms (EPSs), many do not consider the antimuscarinic and histaminergic side effects that can occur with second generation antipsychotics. We present the case of a 62-year-old male who presented with acute urinary retention that was found to be due to the recent initiation of olanzapine. Gradual cessation of the medication and follow-up with urology revealed resolution of his symptoms. With the increasing demand for psychiatric care but limited psychiatric resources, more and more primary care physicians find themselves dealing with the complex challenges of mental illness and managing antipsychotic medications. As a result, coordinating care among multiple specialties and understanding the full profile of side effects that are associated with psychiatric medications can yield quicker diagnoses and improve patient care.

## 1. Background

Acute urinary retention is defined as the inability to pass any urine when the bladder is full despite attempts by the patient which can result in significant pain [1]. Complications of untreated, acute urinary retention include, but are not limited to, infection and renal failure. There are multiple etiologies for acute urinary retention including obstructive, inflammatory, neurogenic, and drug induced. When considering medication-induced urinary retention, anticholinergic medications are a common culprit. Antipsychotics are considered the most efficacious drugs in psychiatry as maintenance therapy of schizophrenia and other acute psychotic disorders such as bipolar I disorder [2]. Two generations exist, with the second generation of antipsychotics, also termed “atypical,” more commonly prescribed in recent years due to their more favorable side effect profile of less EPS [3]. Olanzapine, a second generation antipsychotic, is commonly used in the treatment of bipolar I disorder. While clinicians monitor for the onset of EPS, many do not consider the antimuscarinic and histaminergic side effects that can also occur.

## 2. Case Presentation

A 62-year-old male with a past medical history notable for bipolar I disorder, nephrogenic diabetes insipidus, and metastatic colorectal cancer presented with a chief complaint of nonradiating suprapubic abdominal pain. He had no associated emesis or nausea. He did not note any worsening of his symptoms with oral intake of food. He stated that he was last able to urinate 24 hours prior to presentation. Prior to this, he denied any sexual contacts in the past year and denied urinary frequency out of his baseline. He reported no penile discharge. While he did not have fevers, he did endorse chills and night sweats. He denied any recent acetaminophen use. His abdominal pain continued to worsen over the past 24 hours prompting his presentation to our institution's emergency department.

His past medical history was notable for bipolar I disorder which was previously controlled with lithium for over 30 years but complicated by the subsequent development of nephrogenic diabetes insipidus. He was initially started on lamotrigine 150 mg in the morning 1 year ago and then transitioned to olanzapine 10 mg in the morning 3 months prior to presentation. His medical history is also notable for prior diagnoses of stage IV colorectal cancer, stage 3 chronic kidney disease (CKD stage III), hypertension, and dyslipidemia. His stage IV colorectal cancer was diagnosed approximately a year and a half prior to this admission. He underwent three cycles of palliative chemotherapy with folinic acid, fluorouracil and oxaliplatin, also known as FOLFOX. Treatment was complicated by the development of bacterial colitis and sepsis requiring laparotomy and segmental resection of the colon. He was able to later restart FOLFOX and received 6 additional cycles as well as palliative chemotherapy; however, follow-up computed tomography (CT) and carcinoembryonic antigen levels revealed progression of his metastatic disease and the patient elected to stop further interventions.

Surgical history was notable for hemicolectomy approximately 18 months ago as mentioned above as well as an appendectomy as a teenager. Medications on admission included amlodipine 10 mg daily, losartan 25 mg daily, lovastatin 20 mg daily, lamotrigine 150 mg in the morning, and olanzapine 10 mg in the morning. He denied any drug allergies. Socially, he denied ever smoking tobacco or illicit drugs. He did admit to social alcohol use. He is currently divorced and lives with his son.

On examination, initial vital signs revealed a blood pressure of 154 over 66 mmHg, heart rate of 132 beats per minute, respiratory rate of 16 breaths per minute, normal oxygen saturation of 97% on room air, and a temperature of 97.6 degrees Fahrenheit. He appeared in mild distress. He made appropriate eye contact and was conversing in full sentences. Cardiac exam revealed normal S1 and S2 heart sounds without S3 or S4. He had no appreciable murmurs. Pulmonary examination was unremarkable. Abdominal exam was notable for central suprapubic tenderness with a palpable bladder. No costovertebral angle tenderness. No overlying rashes revealed on exposed skin. Neurologic exam revealed no hyporeflexia of the patellar or Achilles reflexes, no muscle rigidity, or dystonia. Psychological exam revealed no excessive talking or pressured speech. He did not appear anxious or irritable. No evidence of akathisia or tardive dyskinesia.

## 3. Investigations

**Figure 1:**
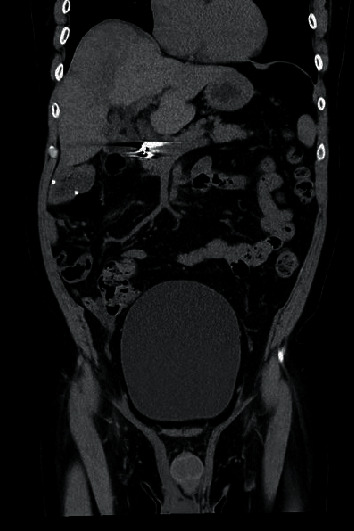
Noncontrast CT abdomen in coronal plane demonstrates enlarged bladder to the level of the umbilicus.

**Figure 2:**
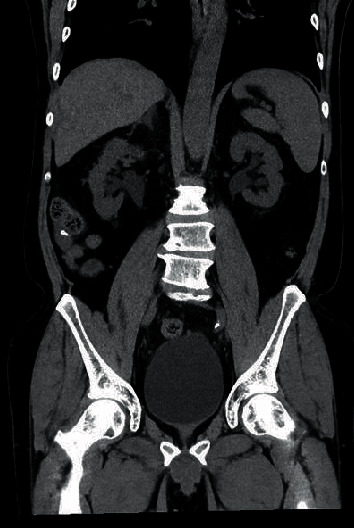
Noncontrast CT abdomen in coronal plane showing bilateral hydronephrosis.

**Figure 3:**
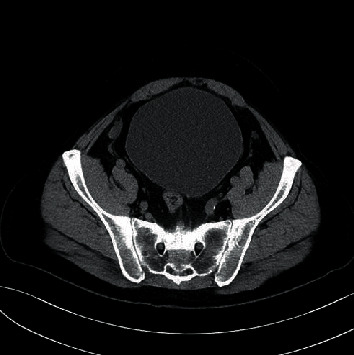
Noncontrast CT abdomen in transverse plane again demonstrates enlarged bladder with no appreciable surrounding enlarged lymph nodes.

Routine blood investigations including complete blood count, renal, and hepatic function tests were performed. Complete blood count revealed a hemoglobin of 13.1 g/dL and a hematocrit of 41.1 g/dL with a mean corpuscular volume of 78.8 fL. Complete metabolic panel was unremarkable except for a mildly elevated sodium of 144 mmol/L, an elevated chloride of 109 mmol/L, and a serum creatinine of 2.21 mg/dL and glomerular filtration rate (GFR), calculated via MDRD, of 30 ml/min/m. Comparison metabolic panel from 1 year prior revealed a serum creatinine of 1.69 mg/dL and an estimated GFR of 41. Urinalysis was colorless with a low specific gravity of 1.002 (consistent with known nephrogenic diabetes insipidus), but no evidence of infection. No proteinuria was evident. Hepatic function tests were within normal limits. Total protein was 7.3 g/dL. Chest X-ray showed no acute cardiopulmonary disease. Given the suprapubic tenderness reported in the setting of acute urinary retention, a CT scan of the abdomen and pelvis without oral or intravenous contrast was obtained. It revealed increasing bilateral hydronephrosis of moderate severity with a full and distended bladder to the level of the umbilicus (see Figures 1–3). The prostate was only mildly enlarged. Right colonic surgical anastomosis consistent with prior colectomy was appreciated but no abnormal lesions within the bladder itself could be appreciated. While it did reveal mildly enlarged mesenteric lymph nodes, no overtly new lesions suggestive of progression of his known stage IV colorectal cancer were visualized.

## 4. Differential Diagnosis

In this patient presenting with acute onset urinary retention in the setting of a distended bladder with moderate bilateral hydronephrosis on imaging with no prior history of bladder or renal conditions, the differential diagnosis is limited to medication side effects, radiation exposure, chemotherapy exposure, metastatic cancer, benign prostate hypertrophy (BPH), and/or infection.

For men presenting with acute urinary retention, one of the first conditions we consider is BPH. Treatment involves immediate bladder decompression with a Foley catheter, as well as consideration of prophylactic pharmacologic treatments to decrease prostatic size such as 5 alpha-reductase inhibitors and alpha blockers [4]. Our suspicion for BPH being the causative factor for his symptoms was weak given imaging showed only mild BPH without evidence of obstructive pathology as well as the acuity of his symptoms, as BPH typically presents slower in onset as progressively weaker voids [5]. The patient had not endorsed any urinary frequency, nocturia, or incomplete bladder emptying more consistent with obstructive pathology.

The patient's chart review and discussion revealed a history of palliative radiation therapy for metastatic disease to the liver. Complications from radiation therapy include strictures of the urethra and ureter tubes [6, 7]. CT imaging did not show evidence of strictures; however, gold standard for diagnosis would include cystourethroscopy, retrograde urethrogram, voiding cystourethrogram, or ultrasound urethrography. However, the discussion of this with urology did not warrant further diagnostic evaluation.

As above, the patient's history of metastatic cancer could have contributed to his current symptoms in one of two ways: mass effect from new metastasis and/or a side effect from prior therapy. He received several rounds of both palliative radiation and chemotherapy with FOLFOX [8]. Though not common, case reports have shown potential of neurotoxicity by oxaliplatin that can lead to urinary retention [9]. With his last dose of chemotherapy being over 1 year prior to his admission, it was unlikely that this was the cause. When his CT imaging was reviewed for evidence of new metastasis, there was no appearance of any mass effect or lesions that could account for an obstructive etiology. While bladder wall involvement was not noted specifically on CT, a positron emission tomography (PET) scan could help evaluate for increased metabolism within the bladder wall. However, with an unremarkable CT, this was not pursued.

With the above conditions unlikely to have caused the patients acute symptoms, our attention focused on his medication regimen. Review of his home medication list revealed two medications rarely associated with urinary retention: compazine and olanzapine. He reported daily compliance with olanzapine and infrequent use of compazine for nausea with no recent changes in dosing of either of these medications. Review of medication side effects for atypical antipsychotics, specifically olanzapine, shows urinary retention as a rare side effect (<1%) by the manufacturer [10–12].

## 5. Treatment

Based on the results of the CT scan, a Foley catheter was placed in the emergency room, immediately yielding 1.2 liters of clear yellow urine. Over the following 30 minutes, an additional 200 cc of urine was voided. The patient was admitted to our service for further care. Review of his medications revealed that he was recently started in the past 3 months on olanzapine. While rare, its anticholinergic properties suggested a possible cause and effect relationship between the medication and the patient's presentation. After consulting with psychiatry, as well as his outpatient psychologist, olanzapine was decreased from 10 mg to 5 mg. The patient remained with a Foley catheter in place, and after consultation with urology, he was started on both tamsulosin 0.4 mg as well as finasteride 5 mg/d. We discussed the CT findings with him and the patient elected not to be seen by an oncologist during this hospitalization but wanted to follow up with them as an outpatient as he did not desire any further treatments. He was discharged 72 hours later with close follow-up planned with his psychologist and oncologist, as well as urology. At discharge, his condition was stable with mild improvement in his abdominal pain, however his persistent pain but was suspected to be secondary to his known underlying malignancy.

## 6. Outcome and Follow-Up

One week after discharge, the patient was seen by his outpatient psychiatrist who stopped olanzapine completely. At the patient's 2-week follow-up with urology, his Foley catheter was removed and he was able to spontaneously void urine without any difficulties later that day. He remains off olanzapine and his bipolar disorder is currently managed with lamotrigine 150 mg daily. He has since followed up with his oncologist and has been placed in palliative care as he does not desire any further treatment. His abdominal pain has improved but has required escalating doses of opiate therapy. As previously mentioned, this is suspected to be secondary to progressive metastasis of known colorectal cancer.

## 7. Discussion

Bipolar disorder is a cyclical mood disorder affecting approximately 1.5% of the population [13]. Patients characteristically present with manic episodes during adolescence or young adulthood, defined as unusual periods of talkativeness, flight of ideas, inflated self-esteem, decreased need for sleep, and impulsivity [14]. In between these episodes of mania are often periods of major depression [9]. It is important to appropriately recognize bipolar disorder in patients presenting with symptoms of major depression but who also have had symptoms consistent with mania or who have a family history of bipolar disorder as initiation of treatment for presumed major depressive disorder with antidepressant therapy can provoke mania and eventual psychosis [15]. Instead of antidepressants, mood stabilizers (lithium, valproic acid, lamotrigine, second generation [“atypical”] antipsychotic drugs, and carbamazepine) are effective for resolving acute episodes of mania, stabilizing depression symptoms, and preventing future flare-ups [16].

Second generation antipsychotic drugs include clozapine (Clozaril), quetiapine (Seroquel), risperidone (Risperdal), and olanzapine (Zyprexa) [17]. They are similar to the first generation antipsychotic class in providing postsynaptic blockade of brain dopamine (D2) receptors however have a more favorable side effect profile, notably less extrapyramidal side effects, due to their greater affinity for the serotonin (5HT2) receptor in contrast to the first generation antipsychotics [18]. Unfortunately, the second generation class also has the potential to bind with *α*-adrenergic and histamine receptors leading to sedation and significant weight gain along with antimuscarinic specific effects such as dry mouth, constipation, and urinary retention [19]. These side effects can have significant effects on tolerability as evidenced by a study from Lieberman et al. comparing differences in overall effectiveness between first generation and second generation antipsychotics in patients with chronic schizophrenia which showed that 74% of the patients taking either first and second generation antipsychotics discontinued their medication before the end of the study secondary to adverse effects [17]. Furthermore, side effects may be more pronounced when initiating a second generation antipsychotic for the first time in a patient. Diurni et al. performed a retrospective review of such patients over a three-month and twelve-month period [20]. Those who were initiated on a second-generation antipsychotic were more likely to experience metabolic effects; however, there were interestingly no significant findings associated with antimuscarinic properties.

Olanzapine is a second generation antipsychotic drug considered first line monotherapy for both acute mania and for maintenance of symptoms. Of the atypical antipsychotics, olanzapine and clozapine have been shown to have the greatest antimuscarinic effects leading to more reported anticholinergic effects than others within the class [21]. Cohen et al. reported acute urinary retention in two elderly patients after being started on olanzapine for psychiatric conditions in The American Journal of Geriatric Pharmacology [22]. While the patients had multiple factors that could lead to urinary retention (i.e., BPH and age), it was not until they had started low dose olanzapine did acute urinary retention with acute kidney injury develop [22]. In a second case report, a 30-year-old female developed acute urinary retention after starting olanzapine for atypical psychosis that resolved once the medication was discontinued and the patient was changed to aripiprazole [23]. Despite its known antimuscarinic effect, most providers watch for the development of extra pyramidal symptoms rather than the lesser known side effects such as acute urinary retention that can come with starting the atypical antipsychotic olanzapine [24]. As such, appropriate diagnosis and treatment is often delayed, leading to inappropriate testing and the addition of more medications in an attempt to ameliorate symptoms.

Our case of a patient diagnosed with bipolar I disorder who was started on olanzapine with slow increase in his daily dosage and subsequently developed acute urinary retention highlights the importance of a thorough medical history, coordination of care among inpatient and community providers, detailed home medication review and knowledge of associated common, and rare, adverse effects of medications. While our patient had other confounding variables which could contribute to his presentation such as mild BPH, side effects from prior chemotherapy treatments, and known metastatic cancer, further discussion with both the patient and his outpatient psychiatrist yielded the recent addition of olanzapine months prior to this admission. This etiology was further supported with subsequent down titration and cessation leading to complete resolution of symptoms.

As reviewed, olanzapine can lead to urinary complications due to binding with peripheral muscarinic receptors in the genitourinary system which interferes with micturition. In a population based, a retrospective cohort study of older adults in Ontario, Canada, it was observed that patients started on atypical antipsychotics were associated with a higher 90-day risk for hospitalization with acute kidney injury likely in part to secondary events such as hypotension and acute urinary retention. Though rare, it is important for both inpatient and outpatient providers to recognize this potential complication to prevent delays in diagnosis and treatment. All providers, not simply those within the psychiatric specialty, should be aware of the myriad of side effects associated with atypical antipsychotics to include the antimuscarinic effects such as acute urinary retention which may induce acute symptoms and eventual hospital admission.

## 8. Learning Points/Take Home Messages

- Acute urinary retention or the complete inability to voluntarily pass urine should be managed with the insertion of a catheter and concurrent initiation of pharmacotherapy blocking alpha receptors along with close follow-up with urology
- While much focus on atypical antipsychotics is on their potential for extrapyramidal side effects, their antimuscarinic effects can lead to adverse outcomes in patients as well [25]
- In patients with multiple medical problems, coordination among multiple treating specialties is essential to ensure that all differentials are considered and a cohesive treatment plan can be rendered [26]
